# COVID-19: Association of risk classification with the Modified Early Warning Score and hospital outcomes

**DOI:** 10.1590/1518-8345.6666.3978

**Published:** 2023-09-18

**Authors:** Alexandra Emidio Neiman, Cássia Regina Vancini Campanharo, Maria Carolina Barbosa Teixeira Lopes, Luiz Humberto Vieri Piacezzi, Ruth Ester Assayag Batista

**Affiliations:** 1 Universidade Federal de São Paulo, Escola Paulista de Enfermagem, São Paulo, SP, Brasil.; 2 Universidade Federal de São Paulo, Escola Paulista de Enfermagem, Departamento de Enfermagem Clínica e Cirúrgica, São Paulo, SP, Brasil.

**Keywords:** Early Warning Scores, MEWS, Risk Assessment, Nursing, Emergency, COVID-19, Señales de alerta temprana, MEWS, Medición de Riesgo, Enfermería, Emergencia, COVID-19, Escores de Alerta Precoce, MEWS, Classificação de Risco, Enfermagem, Emergência, COVID-19

## Abstract

**Objective:**

to evaluate the association of the risk classification categories with the Modified Early Warning Score and the outcomes of COVID-19 patients in the emergency service

**Method:**

a crosssectional study carried out with 372 patients hospitalized with a COVID-19 diagnosis and treated at the Risk Classification Welcoming area from the Emergency Room. In this study, the patients’ Modified Early Warning Score was categorized into without and with clinical deterioration, from 0 to 4 and from 5 to 9, respectively. Clinical deterioration was considered to be acute respiratory failure, shock and cardiopulmonary arrest

**Results:**

the mean Modified Early Warning Score was 3.34. In relation to the patients’ clinical deterioration, it was observed that, in 43%, the time for deterioration was less than 24 hours and that 65.9% occurred in the Emergency Room. The most frequent deterioration was acute respiratory failure (69.9%) and the outcome was hospital discharge (70.3%).

**Conclusion:**

COVID-19 patients who had a Modified Early Warning Scores > 4 were associated with the urgent, very urgent and emergency risk classification categories, had more clinical deterioration, such as respiratory failure and shock, and evolved more to death, which shows that the Risk Classification Protocol correctly prioritized patients at risk of life.

## Abstract (es)

**Objetivo:**

evaluar la asociación entre las categorías de clasificación de riesgo y el Modified Early Warning Score y los resultados de los pacientes con COVID-19 en el servicio de emergencia

**Método:**

estudio transversal, realizado con 372 pacientes hospitalizados con diagnóstico de COVID-19 atendidos en la Recepción con Clasificación de Riesgo en Urgencias. En este estudio, el Modified Early Warning Score de los pacientes se clasificó como sin y con deterioro clínico, de 0 a 4 y de 5 a 9, respectivamente. Se consideró que había deterioro clínico cuando presentaban insuficiencia respiratoria aguda, shock y paro cardiorrespiratorio.

**Resultados:**

el Modified Early Warning Score promedio fue de 3,34. En cuanto al deterioro clínico de los pacientes, se observó que en el 43% de los casos el tiempo de deterioro fue menor a 24 horas y que el 65,9% ocurrió en urgencias. El deterioro más frecuente fue la insuficiencia respiratoria aguda (69,9%) y el resultado fue alta hospitalaria (70,3%).

**Conclusión:**

los pacientes con COVID-19 que presentaban Modified Early Warning Score 4 se asociaron a las categorías de clasificación de riesgo urgente, muy urgente y emergente y tuvieron más deterioro clínico, como insuficiencia respiratoria y shock, y murieron, lo que demuestra que el Protocolo de Clasificación de Riesgo priorizó correctamente a los pacientes con riesgo vital.

## Abstract (pt)

**Objetivo:**

avaliar a associação das categorias de classificação de risco com o Modified Early Warning Score e os desfechos dos pacientes com COVID-19 no serviço de emergência

**Método:**

estudo transversal, realizado com 372 pacientes internados com diagnóstico de COVID-19 atendidos no Acolhimento com Classificação de Risco no Pronto-Atendimento. Neste estudo, o Modified Early Warning Score dos pacientes foi categorizado em sem e com deterioração clínica, de 0 a 4 e de 5 a 9, respectivamente. Foram consideradas deteriorações clínicas a insuficiência respiratória aguda, choque e parada cardiorrespiratória.

**Resultados:**

o Modified Early Warning Score médio foi de 3,34. Em relação à deterioração clínica dos pacientes, observou-se que em 43% o tempo para deterioração foi menor de 24 horas e que 65,9% delas ocorreu no pronto-socorro. A deterioração mais frequente foi a insuficiência respiratória aguda (69,9%) e o desfecho foi o de alta hospitalar (70,3%).

**Conclusão:**

pacientes com COVID-19 que tiveram Modified Early Warning Score 4 foram associados às categorias da classificação de risco urgente, muito urgente e emergente e tiveram mais deterioração clínica, como a insuficiência respiratória e o choque, e evoluíram mais a óbito, o que demonstra que o Protocolo de Classificação de Risco priorizou corretamente os pacientes com risco de vida.

**Highlights:**

**(1)** Association between Modified Early Warning Score and Risk Classification

**(2)** Demonstration of the accuracy of risk classification in prioritizing critically-ill patients.

**(3)** Increased safety through proper stratification of critically-ill patients.

## Introduction

Emergency Services (ERs) provide care for sudden and acute clinical conditions with severe clinical conditions that require urgent intervention, with the objective of stabilizing the patients, preventing deterioration of their conditions and reducing morbidity and mortality ^(^ 1 ^)^. 

In recent decades, the demand for assistance in ERs has increased considerably, having a multifactorial cause and with the possibility of dividing it into those related to the patients, to assistance and to the health system. Among them we can mention growth of the aged population, increase in chronic non-communicable diseases and de-structuring of basic health care, which exert a direct impact on overload of these services ^(^ 2 ^)^. In this context, it was necessary to implement strategies to prioritize care for those individuals at imminent risk of death ^(^ 3 ^)^, as many patients who seek these services have a low risk of death. In this way, through Ordinance 2,048/2002, the Ministry of Health recommended implementing welcoming with Risk Classification (RC) ^(^ 4 ^)^. 

Risk classification protocols aim at systematizing the evaluation, with the objective of prioritizing care according to care urgency through the clinical evaluation of the patients subsidized by the assessment of the main complaints and the signs and symptoms presented by the patients ^(^ 5 ^)^. Although their use in ERs has been adopted since 2004 in Brazil ^(^ 5 ^)^, the protocols applied vary according to the institution and some develop their own RC protocol, which may result in subjectivity in the evaluations. In this context, early warning scores have been incorporated into RC to increase reliability and improve the effectiveness of the evaluations and patient safety ^(^ 6 ^)^. 

Early Warning Scores (EWSs) are values generated from each patient’s physiological data that are routinely recorded and monitored. These scores contribute to the early identification of clinical deterioration in patients, which directly favors objective, fast and effective decision-making, which can directly impact the patients’ outcomes ^(^ 7 ^)^. The Modified Early Warning Score (MEWS) is an EWS that has been used in clinical practice and considers the parameters of systolic blood pressure, heart rate, respiratory rate, body temperature and level of consciousness ^(^ 8 ^-^ 9 ^)^. 

Determination of the scores also contributes to care optimization, as it stratifies the potential deterioration risk, which makes it possible to establish an individualized care plan with emphasis on the need for the re-evaluation of each patient. Added together, the scores establish different risk degrees. Scores above zero require an increase in the frequency of monitoring the patients’ vital signs( ^(^ 9 ^)^. 

A number of studies show that applying the MEWS score in Emergency Rooms results in a positive impact on the patients’ outcomes since, in most cases, it was possible to intervene early in time, before deterioration of their clinical status, with a reduction in complications and deaths ^(^ 9 ^-^ 10 ^)^. 

We are currently living in the midst of the COVID-19 pandemic, a disease caused by the SARS-CoV-2 virus that belongs to the coronavirus family, which causes respiratory infections. COVID-19 can be asymptomatic or symptomatic, with fever, cough and difficulty breathing. Dyspnea may progress to severe respiratory distress syndrome, for which the patient will require ventilatory support( 11). As it is a new disease, easily transmitted and with a risk of severe pulmonary impairment, it has increased the demand for assistance in ERs ^(^ 12 ^-^ 13 ^)^. 

Thus, the importance of implementing clinical deterioration scales, such as MEWS, is highlighted, which allows, in a systematic way, evaluating and anticipating problems with the possibility of instituting early measures, in order to improve the patients’ clinical outcomes. From this perspective, the MEWS scale can contribute to risk classification in Emergency Services, in order to categorize patients with COVID-19 more assertively, to improve care quality and to increase patient safety. In this context, analyzing whether the Risk Classification Protocol used in the institution’s Emergency Service is an adequate tool to assess care urgency for this population with suspected or confirmed COVID-19 diagnoses is important for maintaining health safety during this pandemic. This study has the hypothesis that, among patients classified in the categories with greater care urgency, there will be a greater proportion of patients with MEWS > 4. Additionally, there will be a higher proportion of patients with MEWS < 4 in the categories with less care urgency. Therefore, it is intended to evaluate the association of risk classification categories with the Modified Early Warning Score, clinical deterioration and the outcomes of patients with COVID-19 treated at the emergency service.

## Method

### Study design

A cross-sectional and retrospective study with a quantitative approach.

### Locus

The study was carried out in an Emergency Care Unit ( *Unidade de Pronto Atendimento*, UPA) located in the South area of the city of São Paulo. During the pandemic, the UPA was a reference for the care of COVID-19 patients in the Unified Health System ( *Sistema* Único *de Saúde*, SUS). The Service was adapted with red and orange rooms for urgencies and emergencies, in addition to the creation of the Respiratory Failure Unit ( *Unidade de Insuficiência Respiratória*, UIR), to care for suspected or confirmed COVID-19. The UPA had a care team made up of nurses, nursing technicians, physicians and social workers. 

### Population

The study population consisted of all hospitalized patients with a confirmed COVID-19 diagnosis through the reverse transcription examination followed by polymerase chain reaction (RT-PCR, Reverse Transcription Polymerase Chain Reaction), performed at the UPA or in an external Service, aged at least 18 years old, treated at the Welcoming with Risk Classification Sector during the study period, and who had all the variables recorded in the medical chart for MEWS calculation MEWS, totaling 372 patients.

Risk classification was carried out by nurses, who used an institutional protocol based on the Ministry of Health guidelines. This protocol consists of five categories identified by colors, where each color assumes a recommended waiting time for the patient to be seen by the physician according to care urgency. In the red category, service must be immediate, in the orange category in 10 minutes, in the yellow category in 60 minutes, in the green category in 120 minutes and in the blue category in 240 minutes ^(^ 5 ^)^. Patients in need of urgent care were considered those who were classified in the red, orange and yellow categories; whereas those not in need of urgent care were included in the blue and green categories. 

### Data collection

The data were obtained by the researcher during 2021 from the electronic medical records using a specific instrument, which included sociodemographic variables, risk classification category and physiological parameters such as: body temperature, respiratory rate, heart rate, blood pressure, oxygen saturation, capillary blood glucose and pain; in addition to the main complaint and its duration and personal background.

The patients’ clinical deterioration scores were calculated using MEWS, in which the following parameters are considered: systolic blood pressure, heart rate, respiratory rate, body temperature and level of consciousness (alert, responsive to verbal stimuli, responsive to painful stimuli, unresponsive). Values from 0 to 3 are assigned to each parameter, and the total sum corresponds to the score, ranging from 0 to 13. The higher the score, the greater the clinical deterioration risk and the greater the need for monitoring of vital parameters and clinical evaluation by nurse and physician ^(^ 14 ^)^. Those with MEWS scores > 4 will be considered patients with clinical deterioration, and those with MEWS scores < 4 will be considered as patients without clinical deterioration ^(^ 15 ^)^. 

In this study, Acute Respiratory Failure (ARF), shock and Cardiopulmonary Arrest (CPA) were considered clinical deterioration instances.

### Data treatment and analysis

The statistical analysis was performed using the Statistical Package for the Social Sciences (SPSS) program, version 23. A descriptive analysis was performed; the continuous variables with normal distribution were expressed by calculating their mean and standard deviation values, whereas those without normal distribution were expressed as median, minimum and maximum. Sample calculation was performed for mean values with different variances. Considering the MEWS mean and standard deviation values, we calculated a minimum sample size of 148 *per* group (green/blue and red/orange/yellow), with test power (1-β) – 80% and significance level (α) -5%. 

The association of MEWS with the RC categories was verified by means of the t-test and, when necessary, by resorting to the Mann-Whitney test. To assess the association of MEWS with the clinical outcomes, Analysis of Variance (ANOVA) was used and, when necessary, the Kruskal-Wallis test. The association between the risk classification categories and the clinical outcomes was verified using the Chi-Square test and, when necessary, the Likelihood Ratio Test. In addition, to associate the categorized MEWS (>4 and <4) with the RC categories, time to clinical deterioration and the discharge, death and transfer outcomes, the Mann-Whitney test was used. The significance level considered was 5% (p-value<0.05).

### Ethical aspects

The project was submitted and approved by the Research Ethics Committee of the Federal University of São Paulo, under Certificate of Presentation of Ethical Appreciation ( *Certificado de Apresentação de Apreciação* Ética, CAAE) 32702720.9.00005505. 

## Results

A total of 372 patients were included, who were treated at the UPA Risk Classification from the São Paulo Hospital ( *Hospital São Paulo*, HSP) and had positive results for COVID-19. Most of the study population consisted of men (59%), mean age of 60.78 years old, with Incomplete Elementary School (35.2%), with comorbidities (86%) and categorized as urgent care (orange) (39.8%) ( Table 1). 

**Table 1 -:** Sociodemographic and clinical characteristics of the COVID-19 patients assisted in the Risk Classification are of an Emergency Service. São Paulo, SP, Brazil, 2020

Sociodemographic and clinical variables	Total n (%)
**Age in years old** (n=372)	
Mean (SD*)	60.78 (15.13)
Median	61
Minimum-Maximum	20-98
**Gender** (n=371)	
Female	152 (41)
Male	219 (59)
Not reported	1
**Schooling** (n=284)	
Illiterate	19 (6.7)
Incomplete Elementary School	100 (35.2)
Complete Elementary School	43 (15.1)
Incomplete High School	16 (5.6)
Complete High School	63 (22.2)
Incomplete Higher Education	8 (2.8)
Complete Higher Education	34 (12.0)
Graduate studies	1 (0.4)
Not reported	88
**Comorbidities** (n=372)	
0	52 (14.0)
1	63 (16.9)
2	93 (25.0)
3	77 (20.7)
4+	87 (23.4)
**Risk Classification category** (n=372)	
Blue	1 (0.3)
Green	40 (10.8)
Yellow	107 (28.8)
Orange	148 (39.8)
Red	76 (20.4)

*SD = Standard Deviation

The patients sought the ER a mean of 7.82 days after onset of the symptoms, with 81.2% reporting not having had contact with suspected or confirmed cases. On admission, the mean MEWS was 3.34.

In 43% of the situations, clinical deterioration occurred less than or 24 hours after admission to the institution and, of these, 65.9% were in the Emergency Service. The most frequent deterioration was respiratory failure (RF) (69.9%) and the outcome was hospital discharge (70.3%).

Table 2 presents the association of the risk categories with MEWS and its parameters, type and time for the occurrence of clinical deterioration and outcomes of COVID-19 patients. When associating the risk categories with MEWS, it was observed that patients classified in the blue/green, yellow and orange categories had significantly lower MEWS values when compared to those classified in red (p<0.0001), and these latter had higher prevalence of MEWS > 4 (p<0.0001) ( Table 2). 

With regard to the MEWS parameters, patients in the blue/green category had higher prevalence of respiratory rate (RR) between 15 and 20 breaths *per* minute (brpm) when compared to the other categories. Those in the red category had a higher proportion of RR above 29 brpm (p<0.0023) and alteration in the level of consciousness (p<0.0001) than the others. 

In relation to clinical deterioration, patients in the blue/green, yellow and orange classification showed less deterioration than those in the red category, where the proportion of RF was significantly higher when compared to patients in the other categories (p<0.0051). Patients in the yellow category had a significantly higher proportion of cardiovascular shock than the others (p<0.0326).

Those who presented time for clinical deterioration less than or equal to 24 hours were mostly classified in the red category and, for those in the yellow and orange categories, time to clinical deterioration was greater than 24 hours when compared to the blue/green category (p<0.0001).

In relation to the clinical outcomes, individuals classified in the red category had a lower proportion of hospital discharge and a higher rate of death when compared to the blue/green, yellow and orange categories (p=0.0149).

**Table 2 -:** Association of risk classification categories with Modified Early Warning Score, clinical deterioration, clinical parameters and outcomes (n=372). São Paulo, SP, Brazil, 2020

Variables	Risk Classification				Total	p-value
	Blue/Green n (%)	Yellow n (%)	Orange n (%)	Red n (%)		
**Modified Early Warning Score**						
Mean(SD ^*^)	2.54(1.14)	2.81(1.35)	3.45(1.46)	4.29(1.77)	3.34(1.58)	<0.0001 ^†^
Median(Min-Max)	3(1-5)	3(0-8)	3(1-9)	4(1-9)	3(0-9)	
**Total**	41	107	148	76	372	
**Clinical Risk**						
MEWS ^‡^ >4B	2(2.8)	9(12.5)	30(41.7)	31(43.1)	72(19.4)	
MEWS ^‡^ <4	39(13)	98(32.7)	118(39.3)	45(15)	300(80.6)	<0.000 ^1§^
**Total**	41	100	348	76	372	
**Respiratory rate** (brpm ^||^)						
<15	1(2.4)	7(6.5)	3(2)	1(1.3)	12(3.2)	<0.0001 ^¶^
15 – 20	11(26.8)	20(18.7)	20(13.5)	6(7.9)	57(15.3)	
21 – 29	23(56.1)	63(58.9)	69(46.6)	22(28.9)	177(47.6)	
>29	6(14.6)	17(15.9)	56(37.8)	47(61.8)	126(33.9)	
**Total**	41	107	148	76	372	
**Systolic Blood Pressure** (mmHg ^**^)						
71 – 80	-	1(0.9)	5(3.4)	6(7.9)	12(3.2)	0.0227 ^¶^
81 – 100	2(4.9)	5(4.7)	13(8.8)	10(13.2)	30(8.1)	
>101	39(95.1)	101(94.4)	130(87.8)	60(78.9)	330(88.7)	
**Total**	41	107	148	76	372	
**Level of consciousness**						
Alert (0)	40(97.6)	104(97.2)	131(88.5)	54(71.1)	329(88.4)	<0.0001 ^¶^
Confused (+1)	1(2.4)	1 (0.9%)	14(9.5)	13(17.1)	29(7.8)	
Response to pain (+2)	-	2(1.9)	2(1.4)	5(6.6)	9(2.4)	
Unconscious (+3)	-	-	1(0.7)	4(5.3)	5(1.3)	
**Total**	41	107	148	76	372	
**Time to deterioration**						
≤24 hours	12(29.3)	35(32.7)	60(40.5)	53(69.7)	160(43)	<0.0000 ^§^
>24 hours	8(19.5)	28(26.2)	47(31.8)	15(19.7)	98(26.3)	
No deterioration	21(51.2)	44(41.1)	41(27.7)	8(10.5)	114(30.6)	
**Total**	41	107	148	76	372	
**Final outcome** (n=328)						
Discharge	33(82.5)	77(73.3)	106(71.6)	42(56.8)	258(70.3)	0.0149 ^¶^
Death	4(10)	23(21.9)	34(23)	30(40.5)	91(24.8)	
Transfer	3(7.5)	5(4.8)	8(5.4)	2(2.7)	18(4.9)	
**Total**	40	105	148	74	367	

*SD = Standard Deviation; ^†^Fisher’s Exact Test; ^‡^MEWS = Modified Early Warning Score; ^§^Mann-Whitney Test; ^||^brpm = Breaths *per* minute; ^¶^Chi-Square Test; **mmHg = Millimeters of mercury

## Discussion

MEWS can be considered a multifunctional score and simple to measure, as it uses the patient’s vital parameters as a calculation basis, being able to early detect the need for intervention by the health team ^(^ 15 ^)^. 

In the literature, it is possible to find several worldwide comparisons of MEWS with other early warning scores ^(^ 16 ^,^ 17 ^-^ 18 ^)^; however, national studies that associate this score with the risk classification categories assigned to patients in emergency services are scarce ^(^ 6 ^)^, even though it is widely used in Brazilian private institutions. 

The population of this study had a median age of 61 years old, a fact that can be explained both by population aging and because age is a risk factor for complications related to COVID-19 ^(^ 19 ^)^. The presence of comorbidities, observed in most patients in this sample, can be related to their high mean age. Increased age predisposes to the emergence of comorbidities and the literature points out that their presence contributes to worse outcomes in patients with COVID-19 ^(^ 20 ^)^. 

The majority of the study population was male, which, according to a study carried out by Fiocruz, can be explained by the greater chance of illness and death due to infectious diseases in men when compared to women due to sociocultural and hormonal issues, as female sex hormones reinforce the immune system ^(^ 19 ^)^. With regard to schooling, it was observed that most of the patients had Incomplete Elementary School, a fact that can be associated with the fact that the SUS is a reference especially for the socioeconomically disadvantaged population ^(^ 21 ^)^. 

The most predominant risk classification category was orange, which represents severely-ill patients with a significant risk of evolving to death and requiring urgent care ^(^ 6 ^)^, a clinical condition closely associated with the COVID-19 evolution, which can be rapid and nonspecific ^(^ 21 ^)^. Furthermore, the patients classified in the orange and red categories had higher MEWS scores, indicating that the institutional protocol is adequately classifying patients according to the potential for clinical deterioration. In addition to that, there was an association between the classification obtained and the occurrence of clinical deterioration, noticing that most of the patients classified as orange and red deteriorated in less than 24 hours. A similar result was obtained in a study carried out at a hospital located in northern São Paulo, which aimed at verifying the association between the risk classification, the MEWS score and the clinical outcome of patients who were treated at the urgency and emergency unit. In this study, it was shown that patients classified as urgency and emergency were more hospitalized, which may demonstrate a condition with a greater risk of clinical deterioration ^(^ 6 ^)^. 

As COVID-19 patients are considered more unstable, they are more prone to clinical deterioration, such as the occurrence of respiratory failure in 69.9%, which was observed as deterioration in the study. Another research study, carried out in an international tertiary-level care institution and that included patients with moderate to severe COVID-19 infection, showed a drop in oxygen saturation, which is characterized as an important manifestation of COVID-19 and is related to a worse prognosis ^(^ 18 ^,^ 19 ^,^ 20 ^-^ 21 ^)^. 

In relation to patient deterioration, the mean MEWS in this study was 3.34; in other words, most of the patients did not show clinical deterioration; in addition, 70.3% of the patients were discharged, which indicates lower severity levels. The highest MEWS score (MEWS > 4) was associated with high mortality rates, similar results to those from a retrospective cohort study carried out between 2009 and 2016 in Seoul, South Korea, with patients who presented deterioration alert, whose main objective was to analyze the power of predicting deterioration in the patients’ overall health status and that obtained as a conclusion the fact that early identification scores were able to predict the patients’ mortality ^(^ 22 ^)^. 

Unfavorable clinical outcomes in patients with elevated MEWS scores have also been observed in other studies, such as the retrospective cohort study conducted from January 1 ^st^, 2020, to February 29 ^th^, 2020, at the Hankou Hospital in Wuhan, China, which included 235 patients, among which 37 died and had high MEWS scores ^(^ 24 ^)^. 

In addition to this, another study, carried out with 122 patients at the Ümraniye Training and Research Hospital belonging to the Health Sciences University in Turkey with the purpose of evaluating MEWS, showed that it is an excellent tool for rapid evaluation of patients, with favorable performance in predicting in-hospital mortality ^(^ 23 ^-^ 24 ^)^. 

Although this study was able to show the associations of a high MEWS score with a higher occurrence of clinical deterioration and the association between the 0-4 MEWS interval and a higher discharge outcome rate, some limitations should be highlighted. One of them is that this study was conducted in a single center, which limits generalization of the results. In addition to that, the protocol for Risk Classification used was the institutional one.

In this study, the Risk Classification Protocol properly prioritized the most severe patients with the highest risk of death, according to MEWS. In this way, it was evidenced that MEWS can be a great ally in assessing and determining the care priority degree. In view of the importance of early deterioration scores for the clinical practice and their application, based on parameters easily obtained in the initial evaluation of the patients, it is known that improving these tools is fundamental to increase patient safety during risk classification.

In addition to that, it is extremely important for the clinical practice to find instruments that contribute to an accurate, fast, simple and low-cost evaluation by nurses, based on Risk Classification, helping us to identify patients at risk of clinical deterioration, which may contribute to improving the patients’ outcomes.

## Conclusion

In this study, we concluded that patients classified in the red risk category had higher MEWS scores and higher prevalence of clinical deterioration, mainly in the first 24 hours after admission to the Service, and that death as the most frequent outcome. However, in those classified in the blue/green, yellow and orange categories, the MEWS values were lower, as well as the proportion of clinical deterioration and, in this group, the most frequent outcome was hospital discharge.

## References

[ref-b1] National Health Service (UK) (2018). About urgent and emergency care [Internet].

[ref-b2] Pearce S., Marchand T., Shannon T., Ganshorn H., Lang E. (2023). Emergency department crowding: an overview of reviews describing measures, causes, and harms. Intern Emerg Med.

[ref-b3] Zachariasse J. M., Hagen V., Seiger N., Mackway-Jones K., Veen M., Moll H. A. (2019). Performance of triage systems in emergency care: A systematic review and meta-analysis. BMJ Open.

[ref-b4] (2002). Portaria n° 2048/02 [Internet].

[ref-b5] (2009). Política Nacional de Humanização da Atenção e Gestão do SUS. Acolhimento e classificação de risco nos serviços de urgência [Internet]. http://bvsms.saude.gov.br/bvs/publicacoes/acolhimento_classificaao_risco_servico_urgencia.pdf.

[ref-b6] Mendes T. J. M., Silveira L. M., Silva L. P., Stabile A. M. (2018). Association between reception with risk classification, clinical outcome and the Mews Score. Rev Min Enferm.

[ref-b7] (2020). Empresa Brasileira de Serviços Hospitalares. Escore para alerta precoce [Internet. http://www2.ebserh.gov.br/web/hu-ufjf/escore-para-alerta-precoce.

[ref-b8] Souza B. T., Lopes M. C. B. T., Okuno M. F. P., Batista R. E. A., Goís A. F. T., Campanharo C. R. V. (2019). Identification of warning signs for prevention of in-hospital cardiorespiratory arrest. Rev. Latino-Am. Enfermagem.

[ref-b9] Cipriano E. S. V., Salgado B. S., Oliveira A. N., Aguiar B. G. C. (2018). Implantation of the clinical deterioration score (MEWS) in a private hospital of Rio de Janeiro and its respective results. Enferm Brasil.

[ref-b10] Yazdanyar A., Greenberg M. R., Chen Z., Li S., Greenberg M. R., Buonanno A. P. (2022). A customized early warning score enhanced emergency department patient flow process and clinical outcomes in a COVID-19 pandemic. J Am Coll Emerg Physicians Open.

[ref-b11] Zhang J. J., Dong X., Cao Y. Y., Yuan Y. D., Yang Y. B., Yan Y. Q. (2020). Clinical characteristics of 140 patients infected with SARS-CoV-2 in Wuhan, China. Allergy.

[ref-b12] Janke A. T., Melnick E. R., Venkatesh A. K. (2022). Hospital Occupancy and Emergency Department Boarding During the COVID-19 Pandemic. JAMA Netw Open.

[ref-b13] Kilaru A. S., Scheulen J. J., Harbertson C. A., Gonzales R., Mondal A., Agarwal A. K. (2023). Boarding in US Academic Emergency Departments During the COVID-19 Pandemic. Ann Emerg Med.

[ref-b14] Tavares R. C. F., Vieira A. S., Uchoa L. V., Peixoto  A. A. , Meneseset F. A. (2008). Validation of an early warning score in a pre-intensive care unit. Rev Bras Ter Intensiva.

[ref-b15] Montenegro S. M. S. L., Miranda C. H. (2019). Evaluation of the performance of the modified early warning score in a Brazilian public hospital. Rev Brasil Enferm.

[ref-b16] Hu H., Yao N., Qiu Y. (2022). Predictive Value of 5 Early Warning Scores for Critical COVID-19 Patients. Disaster Med Public Health Prep.

[ref-b17] Aygun H., Eraybar S. (2022). The role of emergency department triage early warning score (TREWS) and modified early warning score (MEWS) to predict in-hospital mortality in COVID-19 patients. Iran J Med Sci.

[ref-b18] Kaeley N., Mahala P., Kabi A., Choudhary S., Hazra A. G., Vempalli S. (2021). Utility of early warning scores to predict mortality in COVID-19 patients: A retrospective observational study. Int J Crit Illn Inj Sci.

[ref-b19] Luo L., Fu M., Li Y., Hu S., Luo J., Chen Z. (2020). The potential association between common comorbidities and severity and mortality of coronavirus disease 2019: A pooled analysis. Clin Cardiol.

[ref-b20] Ge E., Li Y., Wu S., Candido E., Wei X. (2021). Association of pre-existing comorbidities with mortality and disease severity among 167,500 individuals with COVID-19 in Canada: A population-based cohort study. PLoS One.

[ref-b21] Özdemir S., Algın A., Akça H. Ş., Altunok İ., Kokulu K., Eroğlu S. E. (2022). Predictive ability of the MEWS, REMS, and RAPS in geriatric patients with SARS-CoV-2 infection in the emergency department. Disaster Med Public Health Prep.

[ref-b22] Wang L., Lv Q., Zhang X., Jiang B., Liu E., Xiao C. (2020). The utility of MEWS for predicting the mortality in the elderly adults with COVID-19: A retrospective cohort study with comparison to other predictive clinical scores. Peer J.

[ref-b23] Ahn J. H., Jung Y. K., Lee JR, Oh YN, Huh J. W. (2020). Predictive powers of the Modified Early Warning Score and the National Early Warning Score in general ward patients who activated the medical emergency team. PLoS One.

[ref-b24] Uranga A., Villanueva A., Lafuente I., González N., Legarreta M. J., Aguirre U. (2022). Factores de riesgo de deterioro clínico en pacientes ingresados por COVID-19: estudio caso-control. Rev Clin Esp.

